# Clinical audit of nonoperative anaesthesia time and surgical site infections of canine tibial plateau osteotomy procedures

**DOI:** 10.18849/ve.v10i2.706

**Published:** 2025-04-23

**Authors:** Amy Mann, Ciara Barr

**Keywords:** anaesthesia, non-surgical anaesthesia time, small animal, surgical site infection, tibial plateua ostetomy, tplo

## Abstract

**Aims and objectives:** The aim of this clinical audit was to reduce nonoperative anaesthesia time from 131 minutes to 102 minutes while maintaining surgical site infection rates below 10% for canine tibial plateau osteotomy procedures. Data collected from anaesthesia records and electronic medical records provided the data to measure these aims.

**Background:** Increased anaesthesia time increases the risk for perianaesthetic complications including surgical site infections. Through anaesthesia record analysis we recognised that nonoperative anaesthesia time was a variable that could be targeted for improvement. Nonoperative anaesthesia time is defined as total anaesthetic time minus surgical time.

**Methods:** Review of 40 total anaesthetic records (24 initial audit, 16 re-audit) and electronic medical records provided the data we recorded to determine anaesthesia time information and surgical site infection information. Manual audits were conducted to create a value stream map and nonoperative anaesthesia times were analysed for special cause variation utilising statistical process control.

**Results:** Nonoperative anaesthesia time was 131 minutes, roughly half of total anaesthesia time (270 minutes), while surgical site infection rate was 8.3%.

**Implementation of changes (team discussion & changes made):** A new communication protocol was trialed between anaesthesia and surgical teams.

**Re-audit:** The same procedure and analysis were used for the initial audit and re-audit. During the re-audit nonoperative anesthesia time was reduced to 109 minutes and surgical site infection rate was 6.3%.

**Application:** The communication protocol trialed during the re-audit is still in practice. This audit can be applied to other practices looking to better analyse their anaesthetic time variables and to re-evaluate communication procedures for better patient outcomes.

## Introduction

Increased anaesthesia duration increases the incidence of perianaesthetic complications, such as hypothermia, hypotension, bradycardia, hypoventilation, and pain (Gruenheid et al., 2018). The knowledge of these risks, the potential to minimise them, and the improvement of patient outcomes prompted an audit of tibial plateau osteotomy procedures (TPLOs), a common yet time consuming procedure performed at our institution. Anaesthesia time for TPLOs performed at our institution was found to be up to twice as long as other institutions (Cook et al., 2010). Increased anaesthesia time not only impacts patient outcomes but also client satisfaction and institutional profitability as fewer cases can be accommodated per day.

Canine patients undergoing TPLOs are at heightened risk for surgical site infections associated with increased anaesthesia time (Nazarali et al., 2014). Surgical site infections can become time consuming, painful, and costly complications that once again negatively impact patient outcomes and client satisfaction. Therefore, anaesthesia efficiency is vital to ensuring positive patient outcomes following TPLO procedures. In this audit we define total anaesthesia time as the time recorded from patient intubation to extubation. Nonoperative anaesthesia time is defined as total anaesthesia time minus surgical time. We sought to decrease nonoperative time specifically, as this is non-value-added time in which the patient is not having surgery performed and accounts for roughly half of total anaesthesia time.

By performing real time detailed observations of TPLOs and analysis of anaesthesia records from TPLO procedures we sought to identify areas that led to increased nonoperative anaesthesia time. We determined that an average of fifty-seven minutes of nonoperative time, was of non-value added time and this is the parameter we used to set the goals of this audit. Once these areas were identified strategies were tested to reduce the nonoperative anaesthesia time, which resulted in an overall decrease in total anaesthesia time. Patient medical records were also used to record data pertaining to surgical site infection occurrence following TPLO procedures. We accounted for causes of surgical site infections other than increased anaesthesia time such as patient history of pyoderma and client-patient noncompliance. The surgical site infection data was used as a secondary outcome metric to see if these infection rates decreased as the strategies to decrease nonoperative anaesthesia time were tested. We aimed to reduce our nonoperative anaesthesia time from 131 minutes to 102 minutes as it would account for 50% reduction in non-value added time and would represent financial savings to our clients. Our second aim was to maintain our surgical site infection rates below 10%, as the initial infection rate was found to be 8.3%.

## Methods

**Figure 1:**
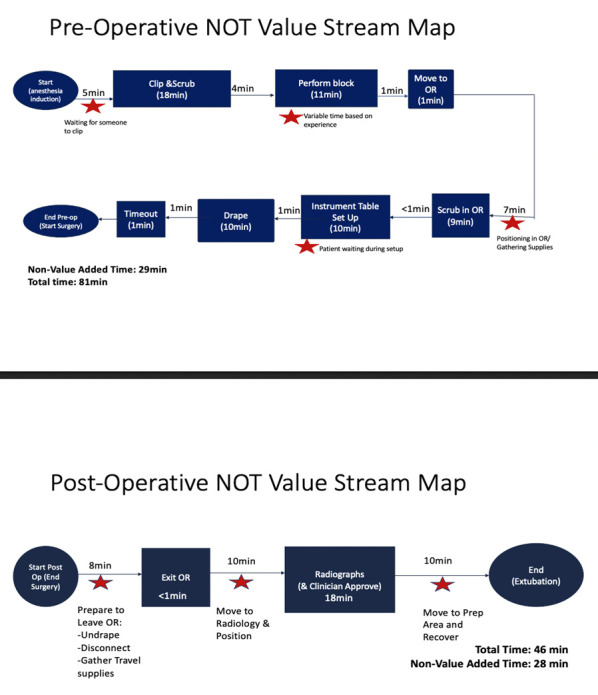
Value stream map of TPLO pre-operative and postoperative nonoperative time (NOT). 57 total minutes of non-value-added time were averaged based on detailed observations during the pre-operative and postoperative periods.

**Figure 2:**
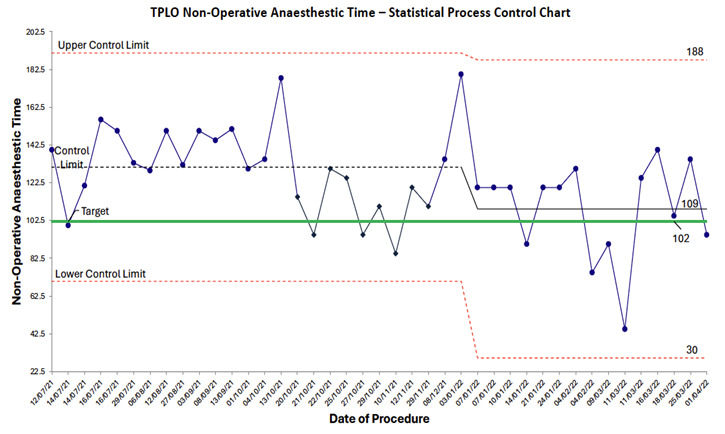
TPLO Nonoperative Time July 2021–April 2022. This statistical process control chart measures nonoperative anesthesia time for TPLOs over the course of the audit and shows a decrease in nonoperative time from 131 min to 109 min following implementation of a structured communication protocol. Target line indicated in green. Control limit represents mean of points in each audit.

40 total cases were included in this audit (24 in the initial audit, 16 in the re-audit). 24 TPLO cases beginning in July 2021 through November 2021 were audited with detailed review of anaesthetic records that included: induction time, surgical start time, surgical end time, and extubation time. Nonoperative time was defined as total anaesthetic time (extubation time minus induction time) minus surgical time (surgical end time minus surgical start time). Detailed manual audits were conducted by trained observers on a convenience sample of 21 cases to create a value stream map which included induction time, waiting time, clipping time, locoregional block time, operating room preparation time, postoperative radiograph time, and recovery time (Figure 1). The nonoperative anesthesia times were analysed for special cause variation during the baseline and intervention time periods utilising statistical process control (Figure 2) (Benneyan et al., 2003). Statistical process control uses statistical significance tests to present time series data in graphic representation. The control chart is used to distinguish between common and special cause variation. The upper and lower control limits of this chart are determined by inherent variation in data while the control limit represents the mean (Benneyan et al., 2003). The TPLO cases were also reviewed for evidence of surgical site infections as described by Nazarali et al. (2014) based on the Center for Disease Control’s (CDC) National Healthcare Safety Network criteria (Center for Disease Control, 2024). The CDC categorises surgical site infections into three groups: superficial incisional (skin and subcutaneous tissues), deep incisional (deep soft tissues), and organ/space. Superficial incisional infections must occur within 30 days of the procedure, while deep incisional and organ/space infections can occur within 30 days to 1 year of the procedure. Each group has criteria for infection involving the presence of mucopurulent material, bacteria on culture, dehiscence, and need for second procedure. For TPLO patients these types of infections can range from superficial incisional infections treated with triple antibiotic ointment to organ/space infections where implant removal is required. Detailed review of anaesthetic records and electronic medical records were used to collect the following: whether arthroscopy was performed, type of locoregional block performed, presence of surgical site infection, and details pertaining to surgical site infection including use of Elizabethan collar, presence of skin infection prior to surgery, and requirement for secondary surgery. Electronic medical records were reviewed for surgical site infections for up to one year after the TPLO was performed. Electronic medical records reviewed included preoperative orthopaedic evaluations, TPLO surgical reports, TPLO procedure discharge instructions, postoperative orthopaedic evaluations, institutional communication log entries, and evaluations from any other services seen within one year following the TPLO procedure.

## Results

The median nonoperative anaesthesia time for TPLOs performed at our institution was 131 minutes with a surgical site infection rate of 8.3% (2/24 cases) during the initial audit. One of the two cases of surgical site infections was reported to have been licking the surgical site following surgery. We did recognise special cause variation during the initial audit period, with 9 audited cases below the control line which was attributed to the Hawthorne effect and increased awareness of anaesthetic time (McCambridge et al., 2014). However, this did not meet our aim of reducing nonoperative anaesthesia time to 102 minutes and was not sustained (Figure 2). Based on the results of the value stream map (Figure 1) a root cause analysis was conducted, which identified that poor communication led to prolonged nonoperative times because they were non-value added time. During the pre-operative period 29 minutes of nonoperative time was classified as non-valued added. During the postoperative period 28 minutes of nonoperative time was classified as non-value added.

### Implementation of changes (team discussion & changes made)

In discussion with key stakeholders including veterinary anaesthesiologists, orthopaedic surgeons, nurses, and hospital leadership, countermeasures were proposed based on the results of the root cause analysis. Utilising an impact effort matrix, the first intervention that was trialed was improving the current communication protocol. For each TPLO case, prior to induction anaesthesia service personnel would send a notification through the intrahospital messaging system stating that the patient was ready to be induced. This would allow orthopaedic service and operating room personnel to be present and prepared before anaesthesia time officially began. This would decrease nonoperative anaesthesia time that was previously used to organise personnel. This described communication protocol was developed in December 2021 and TPLO cases were reaudited following this intervention from January 2022 through April 2022. A potential barrier for this new communication protocol would be a lack of participation. There are many groups involved in making this protocol successful so if participation was lacking from any of these groups, we would not see the overall change in nonoperative anaesthesia time.

### Re-audit results

The same procedure used to perform the initial audit was utilised in the reaudit. The re-audit took place from January 2022 through April 2022 and included 16 cases. Anaesthetic records, electronic medical records, and statistical process control charts were used to analyse nonoperative anaesthesia time and surgical site infection rate. Following the trial of the new communication protocol, special cause variation was noted with a shift in nonoperative time for TPLOs which decreased to 109 minutes (Figure 2). Only one surgical site infection was reported and was associated with the patient licking the surgical site postoperatively. The overall surgical site infection rate during this time remained below 10% at 6.3% (1/16 cases).

### Conclusion

Nonoperative anaesthesia time was reduced with a simple protocol that demonstrates the importance of effective communication in clinical veterinary medicine. In our root cause analysis, we identified ten factors that contributed to increased nonoperative anaesthesia time. Not all these causes were addressed during this audit cycle because their countermeasures required significant capital investment. However, by identifying areas for communication improvement, the team reduced mean average nonoperative time by 22 minutes. In the initial audit average nonoperative anaesthesia time was 131 minutes and average nonoperative anaesthesia time in the re-audit was 109 minutes indicating a reduction on nonoperative anaesthesia time of 22 minutes. Communication failures are known to contribute to adverse events for surgical patients often due to the transfer of care involved during a surgical procedure (Nagpal et al., 2010). In our audit, a structured, direct line of communication between the anaesthesia and surgical teams reduced nonoperative anaesthesia time which may improve patient outcomes, client satisfaction, and institutional profitability.

Clinically, a 22-minute reduction in nonoperative time allows the patient to be discontinued from anaesthetic agents and extubated sooner. This decreases the amount of time the patient is at risk or affected by anaesthetic complications (Gruenheid et al., 2018). It also allows cases to finish earlier allowing more staff availability and potentially more cases to be accommodated daily. Lastly, dependent upon a hospital’s billing system, this could also decrease treatment charges and improve client satisfaction. The statistical process control demonstrated the 22-minute reduction was due to special cause variation and not due to chance alone; there is a possibility that the Hawthorne effect contributed to the change as we observed in our initial audit. Repeating the audit and monitoring if the results are consistent would help validate the sustainability of the communication intervention.

It would be beneficial to explore the root causes and countermeasures not addressed in this audit. These largely pertained to environmental or equipment factors that would require maximum resources to enact. However, continued tracking of surgical site infections and other important outcome metrics may provide evidence for pursuing intervention for larger countermeasures in the future. One limitation of this audit was the relatively few number of cases (40) and correspondingly rare occurrence of surgical site infections leading to the inability to fully analyse the impact of the interventions on surgical site infections. It was also interesting to note the decrease in nonoperative anaesthesia time that occurred during the initial audit period. During the initial audit nonoperative anaesthesia time decreased from 131 to 120 minutes. This change was insufficient to meet the aims of the study but did demonstrate the possible impact of the Hawthorne effect, whereby the process of being observed may have increased efficiency during anaesthesia for TPLOs (McCambridge et al., 2014).

### Application

The exact communication protocol implemented in this audit cycle may not be applicable to smaller patient care teams. Our audit took place at a multi-service referral hospital where there are more instances of transfer of patient care between personnel. The exact communication protocol we describe may not be necessary for every patient care team. However, all patient care teams could use this audit's structure to identify where communication is lacking in their practices to improve anaesthesia time efficiency. Veterinary medicine curriculums have adapted communication education into their programs, which is integral in preparing future veterinarians for client interactions. The authors of this audit believe continued progress can be made in education regarding communication between veterinary care teams. This audit—and previously published audits—document the importance of communication between patient care teams. For example, the use of safety checklists in surgical settings, a form of clinical care team communication, leads to less errors by fostering open communication within the operating room (Russ et al., 2013). Yet when the checklists are not used appropriately or “when individuals have not bought in to the process” the checklists can negatively impact teamwork (Russ et al., 2013). Veterinary curriculums can ensure that future veterinary professionals are “buying into the process” by educating students on the importance of effective communication and its positive impact on patient outcomes. This audit serves as a reminder of the relationship between communication and anaesthesia efficiency.

### Ethical approval

Ethical approval was not required based on the RCVS guidance as there was no change to routine veterinary practice as part of this clinical audit.

### Informed consent

Informed consent was not required for this clinical audit.

### Acknowledgements

The authors would like to thank all those involved in this clinical audit for their support. We would especially like to acknowledge our clinical scheduler, Casey Bacon for assisting with data acquisition and formatting. We would also like to thank Dr. Giacomo Gianotti and Dr. Jason Syrcle for reviewing our manuscript.

### ORCiD

Amy Mann: https://orcid.org/0009-0000-0928-8774

Ciara Barr: https://orcid.org/0000-0001-7727-7647

### Conflict of Interest

The authors declare no conflicts of interest.
